# Evaluating the Association Between Risk Factors of Obstructive Sleep Apnea with Oral Dysfunction and Lifestyle Behavior in Korean Adults Using Data from the Eighth Cycle of the National Health and Nutrition Examination Survey: A Cross-Sectional Study

**DOI:** 10.3390/healthcare13121448

**Published:** 2025-06-17

**Authors:** Won-Jae Jo, Jung-Min Kim, Eun-Seo Choi, Seung-U Lee, Ju Seok Ryu

**Affiliations:** 1 Department of Rehabilitation Medicine, Seoul National University Hospital, Seoul 03080, Republic of Korea; cwj2725@naver.com; 2 National Traffic Injury Rehabilitation Research Institute, National Traffic Injury Rehabilitation Hospital, Yangpyeong 12564, Republic of Korea; 3 Business Incubation Center, Department of Research Planning, Biomedical Research Institute, Seoul National University Bundang Hospital, Seongnam 13620, Republic of Korea; owljm@snu.ac.kr; 4 Department of Rehabilitation Medicine, Seoul National University Bundang Hospital, Seongnam 13620, Republic of Korea; dmstj3275@gmail.com (E.-S.C.); kkdg1040@naver.com (S.-U.L.); 5 Department of Rehabilitation Medicine, Seoul National University College of Medicine, Seoul 03080, Republic of Korea

**Keywords:** obstructive sleep apnea, risk factors of OSA, oral dysfunction, KNHANES, cross-sectional study

## Abstract

**Background/Objectives**: Research on oral dysfunctions as contributing factors to obstructive sleep apnea (OSA) is needed to prevent and treat OSA. This study aimed to explore the association of OSA with oral dysfunction and examine its impact on nutrient intake, physical activity, and handgrip strength. **Methods**: This cross-sectional study analyzed data from the Eighth cycle Korea National Health and Nutrition Examination Survey (KNHANES, 2019–2021). The OSA group included diagnosed individuals and those over 40 years with symptoms such as snoring, fatigue, or witnessed breathing pauses during sleep. The non-OSA group included individuals not meeting these criteria. Using 1:1 propensity score matching to control for confounders (sex, age, lifestyle factors), 7636 participants were included. Oral dysfunction was assessed based on chewing problems, complaints of chewing discomfort, and speech difficulties. Nutrient intake, physical activity, and handgrip strength were analyzed using the Rao–Scott χ^2^ test, complex sample *t*-test, and complex sample logistic regression. **Results**: The OSA group demonstrated significantly more oral dysfunction elements than the non-OSA group (*p* < 0.001). Higher energy intake was observed in the OSA group, with no significant differences in macronutrient intake. Physical activity levels were similar between groups; however, OSA participants without oral problems had higher handgrip strength (*p* < 0.05). Regression analysis showed increased OSA risk correlated with greater oral dysfunction and lower protein intake. **Conclusions**: This study revealed a strong association between oral dysfunction and OSA risk. Focusing on the assessment and early intervention of oral dysfunctions that influence OSA risk factors may aid in the early detection and prevention of OSA.

## 1. Introduction

Obstructive sleep apnea (OSA) is characterized by cessation of breathing or shallow breathing during sleep that occurs due to complete or partial collapse of the upper airway [1]. The episodes are associated with a reduction in respiratory airflow by more than 90% that lasts for at least 10 s [2]. Decrease in respiratory activity due to OSA leads to decreased blood oxygen saturation, excessive daytime sleepiness, snoring, sleep disorders, and cognitive impairment [1,3,4]. If left untreated for a prolonged period, it can result in complications, such as hypertension, heart disease, and stroke, leading to serious cardiovascular conditions [1,3,4]. In addition, OSA has been reported to impose a substantial socioeconomic burden due to significant healthcare costs and reduced quality of life for patients [5,6]. Related medical expenses tend to increase even before diagnosis and remain elevated long after [5,6]. Nevertheless, many individuals with OSA remain undiagnosed, and early detection remains difficult due to high diagnostic costs, limited accessibility, and nonspecific early symptoms [5]. These diagnostic challenges suggest that current screening methods may be insufficient for identifying all cases of OSA, highlighting the need to investigate additional contributing factors.

Among the various factors contributing to OSA development, obesity is one of the significant factors. Patients with OSA are more likely to be overweight or obese and have higher body mass index, fat mass index, and neck, abdominal, and waist circumference [7]. These individuals reportedly eat foods rich in protein, total fat, total saturated fatty acids, and cholesterol while consuming more energy [8]. However, obesity alone cannot fully explain OSA pathogenesis. Various health-related factors contribute to OSA development, including glucose metabolism disorders, vitamin D deficiency, dental factors such as malocclusion and improper dental contacts, and congenital conditions such as Down syndrome, Prader-Willi syndrome, cleft palate, Treacher Collins syndrome, and other craniofacial abnormalities [9,10,11,12,13,14,15,16,17,18,19,20,21]. These previous studies suggest that OSA arises from a variety of causes. However, most of the existing research has primarily focused on anatomical structures and metabolic factors. Oral and swallowing functions, although closely related to the function of muscles such as the genioglossus muscles involved in the pathophysiological mechanisms of upper airway obstruction, have received limited attention as contributing factors [22,23,24].

The pathophysiological mechanism of sleep apnea, beyond that of obesity, involves the action of the genioglossus muscle. When the tension in the palatoglossus and genioglossus muscles decreases considerably, they shift backward and contact the posterior pharyngeal wall, thereby blocking the airway in the supine position during sleep, which can lead to OSA [22,23,24]. The genioglossus muscle is also related to dysphagia, which is observed in 27.3% patients with moderate-to-severe OSA, in whom premature oral leakage is a common symptom [25,26]. A previous study reported that 15% of patients with OSA had symptoms of dysphagia and that dysphagia was independently associated with OSA symptoms, excessive daytime sleepiness, anxiety, and depression [27]. However, studies that directly investigate the relationship between OSA and oral/swallowing functions are lacking. Furthermore, comprehensive studies analyzing these factors alongside nutritional intake, physical activity, and muscle strength as interconnected contributors to OSA pathophysiology are lacking. To effectively prevent and treat OSA, additional research on other factors that can contribute to sleep apnea is required. From this perspective, handgrip strength has emerged as a promising indicator based on prior evidence demonstrating significant associations between handgrip strength and tongue pressure [28,29]. Thus, handgrip strength can be considered a valid proxy for oral muscle capacity, where the concomitant decline in both oral and general muscle strength may contribute to the pathophysiological mechanisms underlying OSA [28,29,30,31,32]. Anatomical mechanisms leading to impaired oral function may influence lifestyle factors, such as nutritional intake, physical activity, and muscle strength measurable by handgrip, thereby potentially contributing to the pathophysiology of OSA [22,23,24,25,26,27,28,29,30,31,32,33,34]. Given the limited focus on functional and lifestyle factors in previous studies, we sought to explore their potential contributions to the pathophysiology of OSA.

This study is significant in presenting the first comprehensive investigation to analyze oral function, nutritional intake and patterns, physical activity, and muscle strength in relation to OSA using large-scale population cohort data from the Korean population. Therefore, we hypothesized that dysfunction in the pharyngeal and tongue muscles could serve as risk factors for the development of OSA by inducing abnormalities in oral functions. The purpose of this study was threefold. First, the primary objective was to unravel the correlation between OSA and oral dysfunction. Second, we aimed to reveal the correlation between OSA and changes in nutritional intake due to oral and swallowing dysfunctions. Third, we sought to examine changes in muscle strength due to OSA and oral dysfunction and to determine the relationship between lifestyle habits such as physical activity and handgrip strength. These findings are expected to provide evidence for the development of functional screening approaches and personalized comprehensive interventions for OSA prevention and management.

## 2. Materials and Methods

### 2.1. Study Design 

This cross-sectional study used data from the Eighth cycle (2019–2021) of the Korea National Health and Nutrition Examination Survey (KNHANES). The KNHANES is a nationally representative dataset that has received ethical approval from the Institutional Review Board (IRB) of the Korea Disease Control and Prevention Agency (2019:2018-01-03-C-A, 2020:2018-01-03-2C-A, 2021:2018-01-03-3C-A) [35]. It is a national health survey conducted on a representative sample of the population aged 1 year and older residing in South Korea, using interview methods, self-administered questionnaires, and direct physical examinations (e.g., height, weight, BMI, waist and neck circumference, handgrip strength) performed by trained professionals. Examiners included licensed healthcare personnel such as physicians, pharmacists, nurses, and medical technologists or graduates of health-related academic programs, all of whom completed standardized training [36]. Data collection was conducted in mobile examination centers where both interviews and clinical assessments were carried out [35]. The survey is carried out with the informed written consent of the participants, and for those under the age of 18, written consent was obtained from their parents or guardians. However, for this specific study, we used data only from participants aged 40 years and older, as the OSA-related questions in the KNHANES were administered exclusively to this age group. The data provided for research purposes are anonymized to protect the privacy of the participants. Therefore, when researchers use the data retrospectively, it is considered exempt from requiring additional informed consent. Accordingly, further review and approval for the exemption were obtained from the IRB of Seoul National University Bundang Hospital. As the study was exempt from requiring additional informed consent, two approvals were received to add analysis data items (The initial approval IRB No.: X-2305-826-901, approved on 16 April 2023, and the final revised approval number is X-2310-856-901, approved on 21 September 2023). This study was conducted in compliance with the Strengthening the Reporting of Observational Studies in Epidemiology statement (STROBE) guidelines.

### 2.2. Study Participants 

The inclusion criteria were as follows: (1) individuals aged ≥ 40 years who responded to the OSA-related variables; (2) those who provided responses to one or more of the following variables (OSA-related variables): diagnostic status of OSA and the risk factors for OSA—snoring, fatigue, and witnessed breathing pauses during sleep; (3) those who provided responses to oral dysfunction-related items—chewing problems, complaint of chewing discomfort, and speech problems due to oral problems; and (4) those who responded to the weighting variable (oral examination and nutrition survey weight), stratification variable, and clustering variable (survey district number). The exclusion criteria were as follows: (1) individuals aged < 40 years and (2) those with missing or unknown data for all variables, including OSA-related variables, propensity score matching (PSM) variables, oral dysfunction variables, and weighting, stratification, or clustering variables.

**Figure 1:**
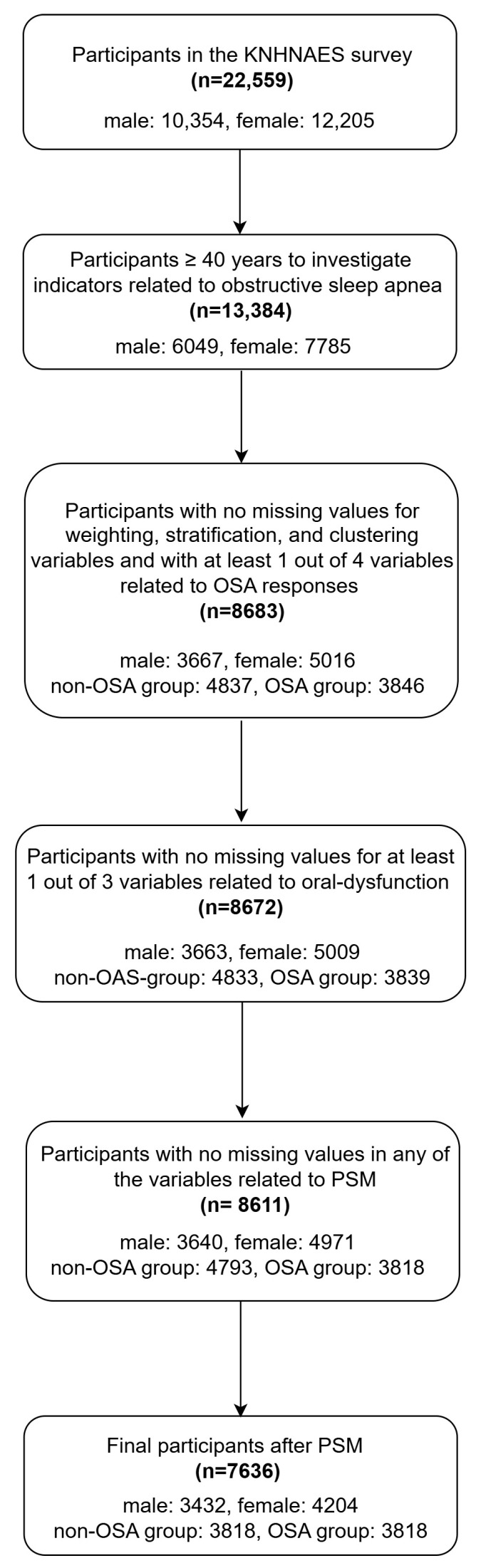
Flow chart for study participants.

The 8th KNHANES data were extracted using two-stage stratified cluster sampling by stratifying the sampling frame based on province, town/village, and housing type. Internal stratification criteria, such as the residential area ratio, age of the household head, and single-person household ratio, were used. Therefore, in this study, we performed nearest-neighbor 1:1 PSM based on a complex sample design that reflected the weighting variable, stratification variable, clustering variable, and PSM variables. Out of 13,834 participants aged ≥ 40 years, 8683 remained after filtering out missing data for key variables such as weighting, stratification, clustering, and OSA-related items. This was further reduced to 8672 after accounting for oral dysfunction data. Among the variables related to PSM, including sex, age, marital status, household income, education, occupation, and other OSA-related factors such as alcohol intake, smoking history, and activity limitation due to dementia, a total of 8611 individuals were selected as the entire study population, excluding any cases with missing values for at least one variable. Using propensity score matching, the final sample comprised 7636 participants. Figure 1 shows a flow chart of the participants of this study. After PSM, the overall mean age was 57.2 ± 11.4 years, with no significant difference between the non-OSA group (57.3 ± 11.5 years) and OSA group (57.0 ± 11.4 years, *p* = 0.30). The mean age of males was 56.7 ± 11.3 years and that of females was 57.7 ± 11.5 years with a significant difference between sexes (*p* = 0.002). Detailed sex- and group- (non-OSA vs. OSA) specific age distributions are shown in Table A1 in Appendix A.

### 2.3. Research Variables

#### 2.3.1. Dependent Variables: Risk of OSA Group

Risk of OSA group (OSA group) comprised participants who responded “yes” to at least one of the four specific items related to OSA. These items included the diagnostic status of OSA and three associated risk factors: snoring, fatigue, and witnessed breathing pauses during sleep. Participants who answered “no” for all four items were categorized into the non-OSA group.

#### 2.3.2. Demographic Characteristics

- PSM Variables: This was applied using demographic factors such as sex, age, marital status, household income, education, occupation, and other OSA-related factors, including alcohol intake, smoking history, and activity limitation due to dementia. Activity limitation due to dementia was identified through a two-step process: participants first answered “yes” to “Do you currently have any limitations in daily life and social activities due to health problems or physical or mental disabilities?” and then selected “dementia” as the reason for their activity limitations in the follow-up question. Age was grouped by decades, and marital status was simplified into categories, such as never married, married (cohabiting), married (separated), and widowed/divorced. Smoking status, determined by the current or past use of various tobacco products, was classified as nonsmoker, current smoker, or ex-smoker.
- Medical History: Medical history was characterized by various medical conditions, including metabolic syndrome (e.g., hypertension, dyslipidemia, diabetes), cerebral disorders (e.g., stroke), cardiovascular disorders (e.g., myocardial infarction, angina), musculoskeletal disorders (e.g., osteoarthritis, rheumatoid arthritis, osteoporosis, gout), respiratory disorders (e.g., pulmonary tuberculosis, asthma, lung cancer, allergic rhinitis, sinusitis), cancer, mental disorders (e.g., depression), and other chronic disorders (e.g., thyroid disorders, atopic dermatitis, etc.).
- Obesity: Participants were categorized into different groups, including underweight, normal weight, overweight, and three obesity classes (Classes 1, 2, and 3), based on the classification defined by the Korean Society for Study of Obesity [37]. Waist circumference was classified using cutoff points of 90 cm for male and 85 cm for female [37]. Neck circumference was categorized according to the STOP-Bang criteria, with a cutoff point of 43 and 41 cm for male and female, respectively [38,39].
- Sleep Duration: Assessment of sleep duration involved the use of the average sleep time on weekdays or weekends. When data were unavailable, sleep duration was calculated by comparing bedtime with wake-up time. Average sleep time was determined as the average value for both weekdays and weekends.

#### 2.3.3. Independent Variables

- Oral Dysfunction: This was analyzed based on specific criteria related to chewing problems, complaints of chewing discomfort, and speech problems due to oral issues. These criteria were adjusted for complex logistic regression analyses, where a score of 5 indicated discomfort, and a score of 1 indicated no discomfort. Individuals experiencing discomfort in one or more of these three variables, such as chewing problems (very uncomfortable or uncomfortable), complaints of chewing discomfort (yes), or speech problems (very uncomfortable or uncomfortable), were classified as having an oral dysfunction.
- Nutrient Intake: Nutrition intake-related variables such as daily energy intake (kcal/day) and macronutrients (g/day) were obtained from the nutrition survey data. The appropriateness of nutrient intake in adults was evaluated based on the 2020 Korean Dietary Reference Intakes [40]. The adequacy of the participants’ energy intake was assessed by comparing their actual intake with the recommended daily energy intake calculated based on their total energy expenditure (TEE). The recommended daily energy intake was tailored based on weight status: an additional 500 kcal for underweight participants, no change for standard weight participants, a reduction of 250 kcal for overweight participants, and a 500 kcal reduction for obese participants (Class I–III). The participants’ energy intake was then categorized as “insufficient” (<75% of recommended intake), “adequate” (75–125%), or “excessive” (>125%).
      Macronutrients were based on the energy content calculated from the participants’ reported daily intake of carbohydrates, proteins, and fats, measured in grams/day. The energy content was calculated using caloric conversion factors: carbohydrates and proteins were multiplied by 4 kcal/g and fats by 9 kcal/g. The calculated energy values were then divided by the total energy, which was the sum of the energy contributions. For carbohydrates, intake was categorized as: “insufficient” if the ratio fell below 55%, “adequate” if it ranged between 55–65%, and “excessive” if the ratio exceeded 65%. Protein intake was classified as “insufficient” when the ratio was below 7%, “adequate” when the ratio ranged between 7–20%, and “excessive” when the ratio surpassed 20%. Finally, fat intake was deemed “insufficient” if the ratio was <15%, “adequate” when it ranged between 15–30%, and “excessive” when it exceeded 30%.
- Physical Activity: The level of physical activity was determined using the Global Physical Activity Questionnaire (GPAQ) [41]. This involved calculating the values of metabolic equivalent of task (METs) from variables related to low-, moderate-, and high-intensity physical activities during work and leisure, as well as walking-related variables (number of days and hours). Using the classification criteria, these values were categorized as low-, moderate-, and high-intensity, leading to the recreation of the GPAQ variable.
- Handgrip Strengths: Handgrip strength data were measured using a digital grip strength dynamometer (T.K.K 5401, Takei Scientific Instruments Co., Ltd., Tokyo, Japan) following the KNHANES protocols. The grip strengths of the left and right hands were measured in triplicate. The average values for each side were calculated, and the grip strength of the hand most frequently used was defined as the handgrip strength. Due to COVID-19 restrictions during 2020–2021, handgrip strength measurements were not available for these years due to insufficient sample sizes from limited regional testing. Therefore, handgrip strength analysis in this study was only based on 2019 data.

### 2.4. Statistical Analysis 

All statistical analyses were conducted using R software version 4.3.1 (R Foundation for Statistical Computing, Vienna, Austria) and RStudio Desktop between June 2023 and May 2024 (results verified with RStudio Version 2024.09.1+394).

- Study Design and Sampling: Complex sampling weights were applied to all analyses using a two-stage stratified cluster design to ensure national representativeness of the Korea National Health and Nutrition Examination Survey (KNHANES) data. The complex survey design incorporated weighting variables, stratification variables, and clustering variables.
- Descriptive Analysis: Categorical and continuous variables were analyzed using the Rao–Scott χ^2^ and complex sample *t*-tests, respectively. Group-wise values for categorical variables were presented as numbers (weighted %) and continuous variables as mean ± standard deviation (SD). Given a large cohort dataset, we assumed normality based on the central limit theorem.
- Multivariate Analysis: Complex sample logistic regression was used for multivariate analysis to assess the impact of various factors on OSA. The analysis comprised five sequential models:
  - Model 1: unadjusted model analyzing oral dysfunction factors (chewing problems, complaint of chewing discomfort, and speech problems)
  - Model 2: Model 1 + nutrient intake variables (energy, carbohydrate, protein, and fat)
  - Model 3: Model 2 + physical activity and handgrip strength (2019 data only)
  - Model 4: Model 3 + PSM variables
  - Model 5: Model 4 + medical history, obesity, waist circumference, and neck circumference

Results from the multivariate analysis were presented as odds ratios (OR) with 95% confidence interval (CI). All tests were based on a significance level of *p* < 0.05.

## 3. Results

Table 1 shows the characteristics of the variables used for propensity score matching (PSM). Table A2 in Appendix A presents the differences in demographics before and after PSM based on these variables.

Table 2 presents the characteristics of participants after PSM, focusing on variables not included in the matching process, such as medical history related to sleep apnea, obesity-related factors, sleep duration, and other known risk factors for OSA. This table highlights baseline differences in health-related characteristics between the OSA and non-OSA groups, providing important context for understanding the study population and interpreting subsequent analyses of oral dysfunction and related outcomes.

**Figure 2:**
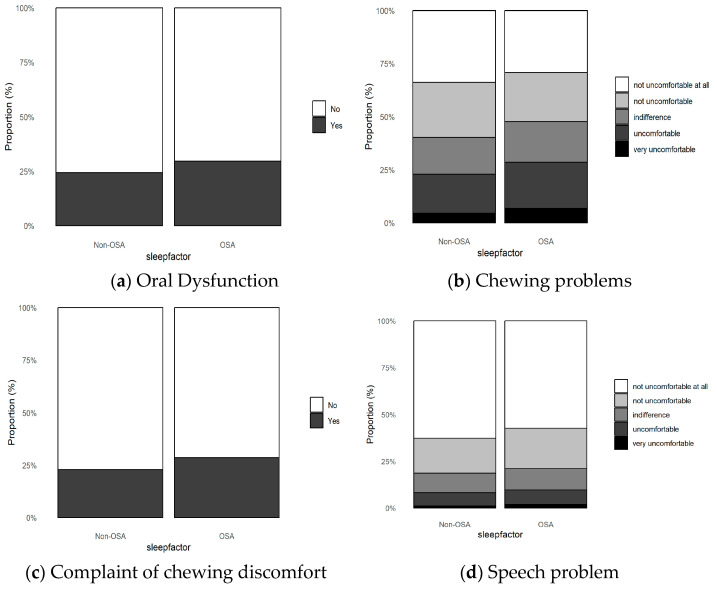
Oral dysfunction problems between the two groups. (**a**). Oral dysfunction prevalence: OSA group showed higher rates (29.6%) compared to non-OSA group (24.4%), including chewing problems, complaint of chewing discomfort, and speech problems (*p* < 0.001). (**b**). Distribution of chewing problems: OSA group showed higher rates (28.5%) compared to non-OSA group (22.9%), including very uncomfortable and uncomfortable categories (*p* < 0.001). (**c**). Complaint of chewing discomfort: OSA group showed higher rates (28.5%) compared to non-OSA group (22.9%) (*p* < 0.001) (**d**). Distribution of speech problems: OSA group showed higher rates of uncomfortable responses (9.7%) compared to non-OSA group (8.3%), including very uncomfortable and uncomfortable categories (*p* < 0.001).

Table 3 and Figure 2 show the differences in variables related to oral dysfunction between the non-OSA and OSA groups. In the context of oral dysfunction such as chewing problems, complaints of chewing discomfort, and speech problems, the OSA group reported a significantly higher proportion of discomfort than the non-OSA group (*p* < 0.001). Moreover, when considering these three variables collectively to form an oral dysfunction variable, the percentage of participants in the OSA group who experienced discomfort was significantly higher (29.6%) than that of the non-OSA group (24.4%).

Table 4 shows no significant differences in nutrient intakes, physical activity, or handgrip strength between the OSA and non-OSA groups, except for a higher energy intake in the OSA group overall and within the non-oral dysfunction subgroup (entire: *p* = 0.003, non-oral dysfunction: *p* = 0.001). The proportions of excessive, adequate, and insufficient energy intake were similar between groups (*p* > 0.05). No significant differences in macronutrient intake were observed (*p* > 0.05). However, the subgroup analysis revealed reduced energy (kcal) and macronutrient intake (protein and fat %) due to oral problems (*p* < 0.001). Handgrip strength was higher in the non-oral dysfunction subgroup (*p* = 0.03) in the OSA group.

Table 5 presents the risk factors of OSA across the five models. Model 1 showed oral dysfunctions to increase by 1.12 times with OSA risk due to chewing problems. Model 2 added dietary factors and demonstrated a 1.12 times increase with chewing problems. Model 3 incorporated physical activity and handgrip, revealing a 1.12 times increase in OSA risk with speech problems, 1.18 times rise with more energy intake. Model 4, adjusted for PSM, indicated a 1.13 times increase with speech problems and a 1.19 times increase with higher energy intake. Finally, Model 5, which took obesity and medical history into consideration, was associated with lower protein intake (odds ratio, 0.06) and higher handgrip strength (odds ratio, 1.05) with an increased OSA risk.

## 4. Discussion

This study aimed to identify the risk factors of OSA and investigate their impact, particularly on oral and chewing problems. In the context of dental issues influenced by the genioglossus muscle, such as chewing problems, complaints of chewing discomfort, and speech problems, the OSA group demonstrated a significantly higher proportion of oral dysfunctions than the non-OSA group (*p* < 0.001). A complex sample logistic regression analysis revealed that chewing and speech problems, higher energy intake, and lower protein intake were all risk factors for OSA, with varying odds ratios across different models. While previous studies on OSA have primarily focused on oropharyngeal dysfunction, this study extends prior findings by directly examining the association between oral dysfunction and OSA using three specific indicators: chewing problems, complaints of chewing discomfort, and speech problems due to oral problems [27,42,43,44,45,46]. Furthermore, by simultaneously considering dietary intake, physical activity, and handgrip strength, we conducted a more comprehensive analysis of the complex interplay between oral function and OSA.

A previous study reported that the prevalence of oropharyngeal dysfunction in patients with OSA ranges from 16% to 78% [1]. In this study, 29.6% of individuals in the OSA risk group showed at least one indicator of oral dysfunction, significantly higher than the 24.4% observed in the non-OSA group (*p* < 0.001). This finding is consistent with previous studies, suggesting that oral and pharyngeal muscles may have a common effect on both oropharyngeal dysfunction and OSA through impaired muscle function [1]. However, no study has directly revealed the relationship between oropharyngeal dysfunction and OSA. Several studies have established the importance of the genioglossus muscle in OSA treatment [27,42,43,44,45,46]. OSA occurs due to a decrease in the tone of upper airway dilator muscles and airway narrowing, which leads to repeated upper airway obstruction caused by an inadequate compensatory response of these muscles. The genioglossus muscle is a major dilator muscle involved in this process [42]. From an anatomical perspective, OSA can occur due to accumulation of fat in the soft tissues and tongue, which leads to narrowing of the upper airway [43]. Previous research on the relationship between OSA and dysphagia revealed that dysphagia treatment can have therapeutic effects in OSA [27]. These effects have been linked to strengthening of the tongue muscles, as well as changes in the upper airway structure [44,45]. This significantly higher prevalence of oral dysfunction in the OSA group (*p* < 0.001) suggests a close association between OSA and oral muscles, highlighting the need for systematic evaluation and preventive intervention targeting oral-related muscular function

Recent therapeutic approaches for OSA primarily target the genioglossus muscle [42]. However, the commonly used STOP-BANG score does not include genioglossus muscle function assessment, which requires specialized equipment [38,39,47,48]. Oral and chewing function evaluation offers an indirect assessment method [1]. Oral dysfunction is not only related to tongue muscle issues, but also to speech problems. Chewing problems often coincide with speech problems. These issues typically arise due to poor oral health, as tooth loss can weaken the support structure of the teeth, leading to difficulties in chewing and speaking [49].

The group with risk factors for OSA had a higher prevalence of metabolic syndrome, musculoskeletal disorders, respiratory disorders, mental disorders, and obesity. Moreover, a relationship between obesity, risk factor for metabolic syndrome, and OSA have been reported [50,51]. Furthermore, in Table 4, an analysis of energy and macronutrient (carbohydrate, protein, fat) intake between the non-OSA group and the OSA group showed that the OSA group had significantly higher energy intake (kcal) compared to the non-OSA group (*p*-value: 0.003). Even within the non-oral dysfunction group, the OSA group had higher energy intake (*p*-value: 0.001). The complex sample logistic regression analysis (Table 5) revealed that despite increased oral dysfunction related to speech problems, energy intake was higher (Model 3: OR 1.18, *p*-value: 0.01; Model 4: OR 1.19, *p*-value: 0.008). However, this can be attributed to incomplete control of obesity, which was found to be a risk factor for OSA in this study. In Model 5, which controlled for obesity-related factors, showed that energy intake (OR: 0.90, *p*-value: 0.9) was not a risk factor for OSA. Additionally, subgroup analysis in Table 4 showed that energy and macronutrients (protein and fat) intake decreased due to oral problems. Previous studies reported that moderate to severe OSA patients showed higher intake of cholesterol, protein, total fat, and saturated fatty acids, even after adjusting for BMI, age, and daytime sleepiness (*p* < 0.05) [8]. However, this study went beyond simple intake comparisons to assess and classify nutrient intake adequacy (insufficient/adequate/excessive) based on total energy expenditure (TEE) and Korean macronutrient ratios (carbohydrate:protein:fat = 55–65%:7–20%:15–30%) from Korean Dietary Reference Intakes [40]. When all OSA-related factors were considered, the OSA risk group showed a higher rate of protein intake deficiency (Model 5: OR 0.06; *p*-value: 0.01). This suggests that oral function deterioration may lead to reduced dietary intake, indicating the need for a comprehensive approach considering oral functional status.

Numerous previous studies have examined tongue muscles, which are important factors involved in the pathophysiological characteristics of sleep apnea syndrome, and their association with nutritional intake [52,53,54]. Studies have shown that the nutrition-related sarcopenia group (NRS group) demonstrated significantly decreased tongue strength (22.9 vs. 29.7 kPa, *p* < 0.001) and lip strength (7.2 vs. 9.9 N, *p* < 0.001) compared to controls, while protein intake >1.2 g/kg/day significantly improved tongue strength (multivariate analysis: 2.613 (0.430, 4.796), *p* = 0.02) [52,53]. Furthermore, several previous studies have reported that nutritional deficiency, assessed using tools such as the Mini Nutritional Assessment (MNA) score, affects tongue strength, particularly showing a high correlation with suprahyoid muscles [54]. In our study, we considered handgrip strength as an indirect indicator of oral-related muscle functions, based on its use in OSA-related muscle strength measurement and sarcopenia determination [28,29,30,31,32,55,56]. However, after adjusting for all covariates, handgrip strength was actually higher in the OSA group, which did not support its significance as an indirect indicator of oral muscle strength. This limitation may be due to the cross-sectional nature of the study.

Complex sample logistic regression analysis (Model 5) revealed that protein intake was a significant indicator. Several studies have reported lower protein intake in groups with chewing- or swallowing-related issues [57,58,59]. Based on previous research, individuals with poor chewing ability showed a significant decrease in protein and fat intake. Patients with chewing problems consumed fewer protein-source foods such as meat, fish, eggs, and dairy products and had reduced dietary diversity [57]. Furthermore, an exploratory study found that dental issues were hindrances to high-protein food consumption [58,59]. Previous studies have shown that improving dietary habits can help individuals with snoring and mild sleep apnea, and that healthy diets can lower OSA risk [60,61]. Consistent with these previous findings, our study also observed a significant correlation between low protein intake and OSA (Model 5: OR: 0.06, *p* = 0.01) [52,53,54,57,58,59,60,61]. While causal relationships cannot be definitively established due to the cross-sectional nature, these findings suggest an interconnected pattern between declined oral function and reduced protein intake, highlighting the need for a comprehensive approach.

Additionally, this study confirmed that the average sleep duration on weekdays in the OSA group (6.6 ± 1.4 h/day) was significantly lower than in the non-OSA group (6.8 ± 1.3 h/day, *p* < 0.001). Similar findings were observed in previous studies examining the impact of sleep duration on OSA patients, where very short sleepers (total sleep time (h) 3.11 ± 0.75, AHI (events/h) 50.18 ± 30.86) had a higher apnea–hypopnea index (AHI) compared to intermediate sleepers (total sleep time (h) 6.37 ± 0.40, AHI (events/h) 20.36 ± 14.68, *p* = 0.007) and sufficient sleepers (total sleep time (h) 7.72 ± 0.56, AHI (events/h) 23.21 ± 20.45, *p* = 0.02) [62]. The AHI, which assesses obstructive sleep apnea based on the number of apneas or hypopneas per hour of sleep, was higher in those with shorter sleep durations [62,63]. This indicates that OSA not only affects the presence of the condition itself but also impacts the quality of sleep (sleep duration) in patients. Beyond sleep duration alone, recent studies have highlighted sleep fragmentation, measured by the arousal index (AI), as an additional clinically meaningful indicator complementary to conventional AHI [64,65]. Sleep fragmentation causes repeated disruptions and triggers excessive sympathetic nervous system activity, thereby increasing the risk of cardiovascular diseases and metabolic syndrome [64]. Studies analyzing hemodynamic parameters have shown that while AHI did not remain significant in regression models, AI remained a robust predictor for both systolic blood pressure (SBP) (β = 0.717, *p* = 0.001) and stroke volume (SV) (β = 0.469, *p* = 0.033), suggesting its potential role as a key clinical marker [65].

The limitations of current diagnostic systems were evident in our study. Despite high rates of self-reported symptoms (fatigue 70.4%, snoring 42.7%, witnessed breathing pauses 19.9%), only 1.2% had received formal diagnosis. This discrepancy, compared to general population prevalence rates of 9–38%, underscores both a lack of recognition of OSA symptoms and challenges in diagnostic accessibility [66,67]. Our results suggest that individuals with risk factors for OSA may not recognize it and do not seek additional testing despite experiencing symptoms such as snoring, fatigue, and witnessed apneas [68]. A definitive diagnosis of sleep apnea requires a polysomnography test, which involves wearing equipment and sleeping in a hospital, making it less accessible to patients owing to the inconvenience involved [69]. Therefore, expanding the analysis of risk factors for OSA and identifying factors that can easily assess the functional status of the genioglossus muscle, which is closely related to OSA, may aid in reducing the discrepancy between OSA risk and the actual diagnostic rate.

In this context, assessing oral function and lifestyle patterns offers several advantages for diagnosing and monitoring the risk of OSA. Recent advancements in home sleep testing devices and wearable technologies have significantly enhanced the accessibility of OSA diagnosis [70,71]. The oral function and lifestyle behavior survey proposed in this study offers multiple benefits in connection with these technologies. Firstly, genioglossus function can be indirectly assessed through oral function evaluations, such as those related to chewing or speaking difficulties, which can offer insights into upper airway patency during sleep [72,73]. Secondly, lifestyle behavior surveys, including dietary intake assessments, are easily collectible and observable in daily life [74]. When linked to OSA status, these surveys can facilitate regular monitoring of OSA risk. Thirdly, these methods are non-invasive and simple screening tools that contribute to the early detection of OSA risks, periodic monitoring, and the development of tailored intervention methods [71,75]. By integrating and analyzing breathing patterns during sleep, as recorded by wearable devices, with oral function evaluation results, it becomes possible to more accurately predict OSA risk and provide personalized management strategies [76,77]. However, to establish the validity and reliability of these methods, further research, such as intervention studies, is necessary beyond the questionnaire-based cross-sectional study approach used in this research [78].

American Academy of Sleep Medicine (AASM) guidelines emphasize individualized treatment strategies for OSA, recommending evidence-based interventions such as oral appliances, weight management, positive airway pressure therapy, and positional therapy. These recommendations reflect a paradigm shift toward personalized assessments and interventions tailored to patient-specific characteristics [79,80,81,82,83]. Accordingly, the oral function and lifestyle-based screening framework proposed in this study aligns with this direction and may contribute to developing a continuous care system for early detection and monitoring of OSA.

In conclusion, assessing oral function and lifestyle behaviors presents a new paradigm for evaluating OSA risk. This approach complements existing diagnostic methods and facilitates early detection and timely, effective management. Through this strategy, we expect to significantly improve the quality of life for OSA patients and provide effective interventions.

### Limitations 

This study had a few limitations. First, this was a cross-sectional study and not a cohort study, which means that it has limitations in terms of confirming causality. Second, data on the size and swallowing function of the genioglossus muscle were not available. Therefore, further analyses using additional data related to swallowing function, which is an important aspect of genioglossus muscle function in patients with OSA may be necessary. Third, we did not control for variables related to oral conditions, such as teeth and gums, or cognitive function, which could be associated with oral dysfunction. Fourth, handgrip strength measurements were only available for 2019 data due to COVID-19-related restrictions, reducing sample size. Moreover, handgrip strength has limitations in indirectly reflecting oral muscular function. Fifth, when we stratified by sex and analyzed the data as shown in Table A1 in Appendix A, we found that despite PSM, age differences between non-OSA and OSA groups persisted within the same sex. This stems from an inherent limitation of PSM, which aims to achieve balance in the overall population but may leave residual imbalances in subgroup analyses. To address this limitation, we performed additional adjustments through multivariate analysis in Table 5. Sixth, although objective measurements (height, weight, BMI, handgrip strength) were conducted by trained investigators, the assessment of OSA symptoms and oral dysfunction relied on self-reported questionnaires, which may compromise reliability, particularly for participants with dementia. Additionally, dementia was identified through self-reported responses to a general activity limitation question rather than clinical diagnosis or standardized assessment tools, which may not accurately reflect clinical status. Further examination for early detection of OSA may be necessary if oral and chewing problems associated with OSA are identified. Lastly, we acknowledge a limitation in our definition of the OSA risk group. We included fatigue as one of the risk factors of OSA, despite it being a non-specific symptom that can result from various health issues unrelated to OSA. Consequently, participants who reported only fatigue, without other characteristic OSA symptoms (such as snoring or witnessed breathing pauses during sleep), were still classified as part of the OSA risk group. This approach may have led to an overestimation of the OSA risk group size by including individuals whose fatigue might not be OSA-related. To address this limitation, future studies might benefit from using more specific criteria or weighting different risk factors to enhance the accuracy of OSA risk classification.

## 5. Conclusions

The results of this cross-sectional study, based on data from the eighth cycle (2019–2021) of the KNHANES, suggest that the risk of OSA is highly associated with oral dysfunction such as chewing problems, complaint of chewing discomfort, and speech problems. A significant relationship between OSA risk and nutrient intake, particularly protein, was also observed. Therefore, evaluation and early intervention for oral dysfunction, which affect the risk factors for OSA, may aid in the early diagnosis and prevention of OSA.

## Figures and Tables

**Table 1:** Demographic Characteristics After Propensity Score Matching.

Variables		Non-OSA (N = 3818)	OSA (N = 3818)	*p*-Values ^2^
		(N, % ^1^)	(N, % ^1^)	
Sex	Male	1697 (44.4%)	1735 (45.4%)	0.5
	Female	2121 (55.6%)	2083 (54.6%)	
Age	Age (Mean ± SD)	57.3 ± 11.5	57.0 ± 11.4	0.3
	40–49	918 (24.0%)	959 (25.1%)	0.8
	50–59	940 (24.6%)	954 (25.0%)	
	60–69	976 (25.6%)	965 (25.3%)	
	70–79	740 (19.4%)	702 (18.4%)	
	≥80	244 (6.4%)	238 (6.2%)	
Marital Status	Never married	170 (4.5%)	131 (3.4%)	0.1
	Married (cohabit)	2879 (75.4%)	2955 (77.4%)	
	Married (separated)	46 (1.2%)	36 (<1%)	
	Widowed/divorced	723 (18.9%)	696 (18.2%)	
Household Income	Lower	682 (17.9%)	734 (19.2%)	0.4
	Lower middle	836 (21.9%)	772 (20.2%)	
	Middle	741 (19.4%)	719 (18.8%)	
	Upper middle	795 (20.8%)	809 (21.2%)	
	Upper	764 (20.0%)	784 (20.5%)	
Education	Elementary school or less	997 (26.1%)	992 (26.0%)	0.4
	Middle school graduate	493 (12.9%)	489 (12.8%)	
	High school graduate	1217 (31.9%)	1156 (30.3%)	
	College or higher	1111 (29.1%)	1181 (30.9%)	
Occupation	Managers, experts, and related worker	385 (10.1%)	443 (11.6%)	0.2
	Office worker	337 (8.8%)	302 (7.9%)	
	Service and sales worker	469 (12.3%)	444 (11.6%)	
	Skilled agricultural, forestry, and fishery workers	182 (4.8%)	213 (5.6%)	
	Technician, machine operator, and assembly worker	407 (10.7%)	416 (10.9%)	
	Simple labor worker	462 (12.1%)	409 (10.7%)	
	Unemployed (housewife, student, etc.)	1576 (41.3%)	1591 (41.7%)	
Alcohol Intake	Non-drinker in the past year	852 (22.3%)	847 (22.2%)	0.2
	Less than once a month	653 (17.1%)	662 (17.3%)	
	About once a month	327 (8.6%)	329 (8.6%)	
	2–4 times a month	699 (18.3%)	640 (16.8%)	
	About 2–3 times a week	485 (12.7%)	556 (14.6%)	
	4 or more times a week	251 (6.6%)	282 (7.4%)	
	Never drank alcohol	551 (14.4%)	502 (13.1%)	
Smoking History	Nonsmoker	2229 (58.4%)	2157 (56.5%)	0.08
	Current smoker	598 (15.7%)	687 (18.0%)	
	Ex-smoker	991 (26.0%)	974 (25.5%)	
Activity Limitation due to Dementia	Yes	3 (<1%)	6 (<1%)	0.4
	No	3815 (99.9%)	3812 (99.8%)	

^1^ Categorical variables: actual numbers (N, weighted %); Continuous variables: weighted mean ± weighted SD, ^2^ Rao–Scott χ^2^ test for categorical, complex sample *t*-test for continuous variables: no significant indicators (*p* > 0.05) for PSM variable post-PSM.

**Table 2:** Characteristics of Participants After Propensity Score Matching: Medical History, Obesity, Sleep Duration, and Obstructive Sleep Apnea-Related Factors.

Variables		Non-OSA (N = 3818)	OSA (N = 3818)	*p*-Value ^2^
		(N, % ^1^)	(N, % ^1^)	
**(1) Medical History**				
Metabolic Syndrome	Present (Yes)	1657 (43.4%)	1868 (48.9%)	<0.001 **
	Past Medical History (Yes)	105 (2.8%)	132 (3.5%)	
	None	2056 (53.9%)	1818 (47.6%)	
Cerebral Disorders	Present (Yes)	84 (2.2%)	105 (2.8%)	0.1
	Past Medical History (Yes)	40 (1.0%)	27 (<1%)	
	None	3694 (96.8%)	3686 (96.5%)	
Cardiovascular Disorders	Present (Yes)	136 (3.6%)	168 (4.4%)	0.1
	Past Medical History (Yes)	24 (<1%)	33 (<1%)	
	None	3658 (95.8%)	3617 (94.7%)	
Musculoskeletal Disorders	Present (Yes)	730 (19.1%)	944 (24.7%)	<0.001 **
	Past Medical History (Yes)	142 (3.7%)	155 (4.1%)	
	None	2946 (77.2%)	2719 (71.2%)	
Respiratory Disorders	Present (Yes)	389 (10.2%)	599 (15.7%)	<0.001 **
	Past Medical History (Yes)	331 (8.7%)	352 (9.2%)	
	None	3098 (81.1%)	2867 (75.1%)	
Cancer	Present (Yes)	103 (2.7%)	113 (3.0%)	0.8
	Past Medical History (Yes)	106 (2.8%)	104 (2.7%)	
	None	2132 (55.8%)	2102 (55.1%)	
	No response	1477 (38.7%)	1499 (39.3%)	
Mental Disorders	Present (Yes)	83 (2.2%)	173 (4.5%)	<0.001 **
	Past Medical History (Yes)	68 (1.8%)	108 (2.8%)	
	None	3667 (96.0%)	3537 (92.6%)	
Others Chronic Disorders	Present (Yes)	507 (13.3%)	712 (18.6%)	<0.001 **
	Past Medical History (Yes)	670 (17.5%)	725 (19.0%)	
	None	2641 (69.2%)	2381 (62.4%)	
**(2) Obesity**				
Body Mass Index (BMI)	BMI (kg/m^2^, Mean ± SD)	23.8 ± 3.2	24.8 ± 3.6	<0.001 **
	Underweight (BMI < 18.5)	119 (3.1%)	80 (2.1%)	<0.001 **
	Normal weight (18.5 ≤ BMI < 23.0)	1479 (38.7%)	1145 (30.0%)	
	Overweight (23.0 ≤ BMI < 25.0)	926 (24.3%)	922 (24.1%)	
	Class 1 Obesity (25.0 ≤ BMI < 30.0)	1116 (29.2%)	1327 (34.8%)	
	Class 2 Obesity (30.0 ≤ BMI < 35.0)	129 (3.4%)	260 (6.8%)	
	Class 3 Obesity (BMI ≥ 35.0)	19 (<1%)	28 (<1%)	
	No response	30 (<1%)	56 (1.5%)	
Waist Circumference	cm (Mean ± SD)	84.2 ± 9.5	87.2 ± 10.1	<0.001 **
	Excessive (Male ≥90 cm, Female ≥85 cm)	1430 (37.5%)	1784 (46.7%)	<0.001 **
	Adequate (Male <90 cm, Female <85 cm)	2379 (62.3%)	2022 (53.0%)	
	No response	9 (<1%)	12 (<1%)	
Neck Circumference	cm (Mean ± SD)	35.1 ± 3.3	35.9 ± 3.5	<0.001 **
	Excessive (Male ≥43 cm, Female ≥41 cm)	32 (<1%)	69 (1.8%)	0.003 *
	Adequate (Male <43cm, Female <41cm)	3772 (98.8%)	3731 (97.7%)	
	No response	14 (<1%)	18 (<1%)	
**(3) Sleep Duration**				
Sleep Duration	Weekday (hr, Mean ± SD)	6.8 ± 1.3	6.6 ± 1.4	<0.001 **
	Weekend (hr, Mean ± SD)	7.2 ± 1.5	7.2 ± 1.6	0.2
	Overall average (hr, Mean ± SD)	7.0 ± 1.3	6.9 ± 1.4	0.003 *
**(4) Obstructive Sleep Apnea Factors**				
The Diagnosis of Obstructive Sleep Apnea	Yes	0 (0%)	46 (1.2%)	<0.001 **
	No	3818 (100%)	3772 (98.8%)	
Risk Factor (1) Snoring	Yes	0 (0%)	1631 (42.7%)	<0.001 **
	No	3815 (99.9%)	2182 (57.2%)	
	No response	3 (<1%)	5 (<1%)	
Risk Factor (2) Fatigue	Yes	0 (0%)	2686 (70.4%)	<0.001 **
	No	3817 (100%)	1132 (29.7%)	
	No response	1 (<1%)	0 (0%)	
Risk Factor (3) Witnessed Breathing Pauses during Sleep	Yes	0 (0%)	760 (19.9%)	<0.001 **
	No	3818 (100%)	3055 (80.0%)	
	No response	0 (0%)	3 (<1%)	

^1^ Categorical variables: actual numbers (N, weighted percentages %); Continuous variables: weighted means ± weighted standard deviations (SD). ^2^ Categorical variables: the Rao–Scott χ^2^ test; Continuous variables: the complex sample *t*-test * *p* < 0.01; ** *p* < 0.001.

**Table 3:** Oral dysfunction according to the two groups.

Factors	Variables	Non-OSA (N = 3818)	OSA (N = 3818)	Rao–Scott F ^3^	*p*-Value ^3^
		N, % ^1^	N, % ^1^		
Oral Dysfunctions ^2^	Yes	931 (24.4%)	1131 (29.6%)	19.34	<0.001 *
	No	2887 (75.6%)	2687 (70.4%)		
Chewing Problems	Very uncomfortable	168 (4.4%)	257 (6.7%)	9.04	<0.001 *
	Uncomfortable	707 (18.5%)	832 (21.8%)		
	Indifferent	661 (17.3%)	728 (19.1%)		
	Not uncomfortable	991 (26.0%)	888 (23.3%)		
	Not uncomfortable at all	1291 (33.8%)	1113 (29.2%)		
Complaint of Chewing Discomfort	Yes	875 (22.9%)	1089 (28.5%)	22.91	<0.001 *
	No	2943 (77.1%)	2729 (71.5%)		
Speech Problems	Very uncomfortable	40 (1.0%)	72 (1.9%)	5.83	<0.001 *
	Uncomfortable	276 (7.2%)	297 (7.8%)		
	Indifferent	397 (10.4%)	438 (11.5%)		
	Not uncomfortable	713 (18.7%)	822 (21.5%)		
	Not uncomfortable at all	2392 (62.7%)	2189 (57.3%)		

^1^ In this study, categorical data were presented as actual numbers (N) with weighted percentages, and the Rao–Scott χ^2^ test was used for association analysis, adjusting for the complex survey design. ^2^ Oral dysfunctions in this study were defined as having one or more of the following issues: chewing problems (categorized as very uncomfortable or uncomfortable), complaint of chewing discomfort (yes), and speech problems (also classified as very uncomfortable or uncomfortable). ^3^ All variables are categorical, so the tests were conducted using the Rao–Scott χ^2^ test. * *p* < 0.001.

**Table 4:** Factors related to obstructive sleep apnea and oral dysfunctions.

Variables	Entire (N = 7636)			Non-Oral Dysfunction (N = 5574)			Oral Dysfunction (N = 2062)			*p*-Value ^1^ (Rao–Scott F) [Between 2 Groups Non-Oral Dysfunction vs. Oral Dysfunction]	
	Non-OSA (N = 3818)	OSA (N = 3818)	*p*-Value ^1^ (Rao–Scott F)	Non-OSA (N = 2887)	OSA (N = 2687)	*p*-Value ^1^ (Rao–Scott F)	Non-OSA (N = 931)	OSA (N = 1131)	***p*-Value** ^1^ **(Rao–Scott F)**	**Non-OSA**	**OSA**
**Nutrient Intakes**											
(1) Energy Intake											
kcal (Mean ± SD)	1821.6 ± 775.9	1889.7 ± 830.6	0.003 **	1849.2 ± 791.0	1934.5 ± 840.1	0.001 **	1726.3 ± 713.4	1767.1 ± 791.5	0.3	<0.001 ***	<0.001 ***
Insufficient	1324 (34.7%)	1254 (32.8%)	0.1 (F = 2.35)	996 (34.5%)	867 (32.3%)	0.1 (F = 1.96)	328 (35.2%)	387 (34.2%)	0.6 (F = 0.55)	0.8 (F = 0.17)	0.4 (F = 0.89)
Adequate	1867 (48.9%)	1838 (48.1%)		1424 (49.3%)	1318 (49.1%)		443 (47.6%)	520 (46.0%)			
Excessive	590 (15.5%)	667 (17.5%)		447 (15.5%)	471 (17.5%)		143 (15.4%)	196 (17.3%)			
No response	37 (1.0%)	59 (1.5%)		20 (<1%)	31 (1.2%)		17 (1.8%)	28 (2.5%)			
(2) Macronutrient Intake											
(2-1) Carbohydrate											
% (Mean ± SD)	63.5 ± 11.2	63.4 ± 11.2	0.6	62.7 ± 11.0	62.7 ± 11.1	0.8	66.3 ± 11.3	65.4 ± 11.1	0.1	<0.001 ***	<0.001 ***
Insufficient	711 (18.6%)	684 (17.9%)	0.7 (F = 0.37)	603 (20.9%)	533 (19.8%)	0.7 (F = 0.37)	108 (11.6%)	151 (13.4%)	0.6 (F = 0.45)	<0.001 *** (F = 22.01)	<0.001 *** (F = 16.72)
Adequate	1129 (29.6%)	1114 (29.2%)		887 (30.7%)	826 (30.7%)		242 (26.0%)	288 (25.5%)			
Excessive	1976 (51.8%)	2019 (52.9%)		1395 (48.3%)	1327 (49.4%)		581 (62.4%)	692 (61.2%)			
No response	2 (<1%)	1 (<1%)		2 (<1%)	1 (<1%)		0 (0%)	0 (0%)			
(2-2) Protein											
% (Mean ± SD)	15.3 ± 4.3	15.5 ± 4.3	0.1	15.5 ± 4.3	15.7 ± 4.3	0.2	14.5 ± 4.2	14.9 ± 4.2	0.1	<0.001 ***	<0.001 ***
Insufficient	20 (<1%)	14 (<1%)	0.7 (F = 0.37)	14 (<1%)	8 (<1%)	0.6 (F = 0.45)	6 (<1%)	6 (<1%)	0.9 (F = 0.09)	0.05 (F = 2.98)	0.07 (F = 2.73)
Adequate	3370 (88.3%)	3377 (88.4%)		2526 (87.5%)	2356 (87.7%)		844 (90.7%)	1021 (90.3%)			
Excessive	426 (11.2%)	426 (11.2%)		345 (12.0%)	322 (12.0%)		81 (8.7%)	104 (9.2%)			
No response	2 (<1%)	1 (<1%)		2 (<1%)	1 (<1%)		0 (0%)	0 (0%)			
(2-3) Fat											
% (Mean ± SD)	21.2 ± 9.1	21.2 ± 9.1	0.9	21.8 ± 9.1	21.7 ± 9.2	0.8	19.2 ± 9.0	19.7 ± 8.8	0.3	<0.001 ***	<0.001 ***
Insufficient	1142 (29.9%)	1189 (31.1%)	0.5 (F = 0.76)	764 (26.5%)	763 (28.4%)	0.2 (F = 1.48)	378 (40.6%)	426 (37.7%)	0.5 (F = 0.63)	<0.001 *** (F = 25.60)	<0.001 *** (F = 15.70)
Adequate	2151 (56.3%)	2088 (54.7%)		1686 (58.4%)	1498 (55.7%)		465 (49.9%)	590 (52.2%)			
Excessive	523 (13.7%)	540 (14.1%)		435 (15.1%)	425 (15.8%)		88 (9.5%)	115 (10.2%)			
No response	2 (<1%)	1 (<1%)		2 (<1%)	1 (<1%)		0 (0%)	0 (0%)			
**Physical Activity**											
METs (Mean ± SD)	765.9 ± 1210.4	813.3 ± 1394.6	0.2	793.4 ± 1199.0	840.5 ± 1413.0	0.3	670.3 ± 1245.2	738.5 ± 1340.5	0.3	0.03 *	0.09
Low Activity	2595 (68.0%)	2608 (68.3%)	0.07 (F = 2.73)	1916 (66.4%)	1783 (66.4%)	0.2 (F = 1.44)	679 (72.9%)	825 (72.9%)	0.1 (F = 2.17)	<0.001 *** (F = 7.62)	0.003 ** (F = 5.85)
Moderate Activity	996 (26.1%)	931 (24.4%)		778 (26.9%)	690 (25.7%)		218 (23.4%)	241 (21.3%)			
High Activity	222 (5.8%)	276 (7.2%)		191 (6.6%)	213 (7.9%)		31 (3.3%)	63 (5.6%)			
No response	5 (<1%)	3 (<1%)		2 (<1%)	1 (<1%)		3 (<1%)	2 (<1%)			
**Handgrip Strengths** ^2^ (Based on the stronger hand among both hands)											
kg (Mean ± SD)	87.8 ± 28.8	89.8 ± 31.3	0.1	89.2 ± 28.6	92.5 ± 31.2	0.03 *	82.5 ± 28.7	82.7 ± 30.5	0.9	0.002 **	<0.001 ***

^1^ Categorical variables, presented as actual numbers (N) with weighted percentages, were analyzed using the Rao–Scott χ^2^ test, while continuous variables, shown as weighted means ± weighted standard deviations, were evaluated with the complex sample *t*-test. ^2^ Handgrip strength analysis was based on 2019 data only due to COVID 19-related measurement restrictions in 2020–2021. * *p* < 0.05; ** *p* < 0.01; *** *p* < 0.001.

**Table 5:** Complex sample logistic regression analysis in obstructive sleep apnea.

	Model 1		Model 2		Model 3		Model 4		Model 5	
	OR ^1^ (95% CI ^2^)	*p*-Value	OR ^1^ (95% CI ^2^)	*p*-Value	OR ^1^ (95% CI ^2^)	*p*-Value	OR ^1^ (95% CI ^2^)	*p*-Value	OR ^1^ (95% CI ^2^)	*p*-Value
Chewing Problems	1.12 (1.03, 1.21)	0.006 **	1.12 (1.03, 1.21)	0.007 **	1.09 (0.96, 1.23)	0.2	1.09 (0.96, 1.24)	0.2	0.57 (0.22, 1.50)	0.3
Complaint of chewing discomfort	1.00 (0.80, 1.24)	>0.9	1.00 (0.80, 1.24)	>0.9	1.09 (0.77, 1.55)	0.6	1.09 (0.77, 1.55)	0.6	2.59 (0.16, 42.10)	0.5
Speech Problems	1.00 (0.94, 1.07)	>0.9	1.01 (0.94, 1.07)	0.8	1.12 (1.01, 1.24)	0.03 *	1.13 (1.02, 1.25)	0.02 *	1.90 (0.78, 4.62)	0.2
Energy Intake			1.08 (1.00, 1.17)	0.05	1.18 (1.04, 1.35)	0.01 *	1.19 (1.05, 1.36)	0.008 **	0.90 (0.26, 3.07)	0.9
Carbohydrate Intake			0.99 (0.88, 1.11)	0.8	1.15 (0.96, 1.38)	0.1	1.16 (0.97, 1.39)	0.1	1.50 (0.30, 7.40)	0.6
Protein Intake			1.07 (0.89, 1.28)	0.5	1.25 (0.93, 1.68)	0.1	1.25 (0.93, 1.69)	0.1	0.06 (0.01, 0.45)	0.01 *
Fat Intake			0.99 (0.87, 1.12)	0.8	1.02 (0.83, 1.25)	0.8	1.01 (0.82, 1.24)	>0.9	2.84 (0.30, 27.09)	0.4
Physical Activity					1.15 (0.99, 1.33)	0.06	1.15 (1.00, 1.34)	0.05	1.58 (0.46, 5.39)	0.5
Handgrip Strengths					1.00 (1.00, 1.01)	0.08	1.00 (1.00, 1.01)	0.8	1.05 (1.00, 1.10)	0.04 *

(1) Model 1: Unadjusted model and analyzed oral dysfunctions (chewing problems, complaint of chewing discomfort, and speech problems). (2) Model 2: Based on Model 1, nutrient intakes (energy, carbohydrate, protein, and fat) were included in the analysis. (3) Model 3: Based on Model 2, the analysis incorporated physical activity and handgrip strengths (2019 data only). (4) Model 4: Adjusted the propensity score matching variable. (5) Model 5: Based on Model 3, adjusted the medical history, obesity, waist circumference, and neck circumference. ^1^ OR = Odds Ratio; ^2^ 95% CI = 95% Confidence Interval; * *p* < 0.05; ** *p* < 0.01.

## Data Availability

All data were obtained from the official KNHANES website—https://knhanes.kdca.go.kr/knhanes/main.do (accessed on 1 June 2023). If you need information about the code, you can request access from the first authors, Won-Jae Jo (cwj2725@naver.com) or Jung-Min Kim (owljm@snu.ac.kr).

## Abbreviations

The following abbreviations are used in this manuscript:

OSA	Obstructive sleep apnea
KNHANES	Korea National Health and Nutrition Examination Survey
IRB	Institutional Review Board
STROBE	Strengthening the Reporting of Observational Studies in Epidemiology statement
PSM	Propensity score matching
TEE	Total energy expenditure
GPAQ	Global Physical Activity Questionnaire
METs	Metabolic equivalent of task
SD	Standard deviation
OR	Odds ratio
CI	Confidence interval
BMI	Body mass index
NRS	Nutrition-related sarcopenia
MNA	Mini nutritional assessment
AHI	Apnea–hypopnea index
AI	Arousal index
SBP	Systolic blood pressure
SV	Stroke volume
AASM	American Academy of Sleep Medicine

## Appendix A

**Table A1:** Distribution of age (Mean ± SD) according to sex and OSA status before and after propensity score matching.

Sex	Overall Series				Propensity Score Matching			
	Overall	Non-OSA	OSA	*p*-Value ^1^	Overall	Non-OSA	OSA	*p*-Value ^1^
All	57.7 ± 11.5	58.2 ± 11.7	57.0 ± 11.4	<0.001 ***	57.2 ± 11.4	57.3 ± 11.5	57.0 ± 11.4	0.3
Male	57.3 ± 11.5	58.4 ± 11.8	56.0 ± 11.1	<0.001 ***	56.7 ± 11.3	57.4 ± 11.5	56.0 ± 11.1	<0.001 ***
Female	58.1 ± 11.6	58.1 ± 11.6	58.1 ± 11.5	0.8	57.7 ± 11.5	57.2 ± 11.5	58.1 ± 11.5	0.02 *
*p*-value ^2^	0.004 **	0.3	<0.001 ***	-	0.002 **	0.6	<0.001 ***	-

^1^ Age differences between Non-OSA and OSA groups were assessed using complex sample *t*-test for the overall population and by sex. After propensity score matching (PSM), no significant age difference was observed between groups in the overall population (*p* = 0.30); however, significant age differences persisted when stratified by sex (*p* < 0.05). ^2^ The table also presents the distribution of age differences between males and females within each groups. In the OSA group, significant sex-based age distribution differences were observed both before and after PSM (*p* < 0.05), with males showing lower mean ages compared to females. * *p* < 0.05; ** *p* < 0.01; *** *p* < 0.001.

**Table A2:** Participant demographics before and after propensity score matching.

Variables	Overall Series				Propensity Score Matching				PBR ^2^
	Non-OSA (N = 4793)	OSA (N = 3818)	SMD ^2^	*p*-Value ^3^	Non-OSA (N = 3818)	OSA (N = 3818)	SMD ^2^	*p*-Value ^3^	
	N, % ^1^	N, % ^1^			N, % ^1^	N, % ^1^			
Sex									
Male	1905 (45.3%)	1735 (52.2%)	−0.06	<0.001	1697 (44.4%)	1735 (45.4%)	−0.01	0.5	82.5
Female	2888 (54.7%)	2083 (47.8%)			2121 (55.6%)	2083 (54.6%)			
Age									
Age (Mean ± SD)	58.2 ± 11.7	57.0 ± 11.4	−0.09	<0.001	57.3 ± 11.5	57.0 ± 11.4	−0.02	0.3	73.7
40–49	1047 (27.5%)	959 (32.0%)	-	<0.001	918 (24.0%)	959 (25.1%)	-	0.8	-
50–59	1160 (30.3%)	954 (30.4%)			940 (24.6%)	954 (25.0%)			
60–69	1239 (21.2%)	965 (19.8%)			976 (25.6%)	965 (25.3%)			
70–79	992 (15.7%)	702 (13.6%)			740 (19.4%)	702 (18.4%)			
≥80	355 (5.4%)	238 (4.2%)			244 (6.4%)	238 (6.2%)			
Marital Status									
Never married	197 (5.1%)	131 (4.3%)	−0.02	0.1	170 (4.5%)	131 (3.4%)	−0.01	0.1	61.2
Married (cohabit)	3615 (78.2%)	2955 (80.6%)			2879 (75.4%)	2955 (77.4%)			
Married (separated)	56 (1.1%)	36 (<1%)			46 (1.2%)	36 (<1%)			
Widowed/divorced	925 (15.5%)	696 (14.2%)			723 (18.9%)	696 (18.2%)			
Household Income									
Lower	847 (13.4%)	734 (14.2%)	−0.02	0.3	682 (17.9%)	734 (19.2%)	<0.01	0.4	84.2
Lower middle	1038 (19.1%)	772 (17.4%)			836 (21.9%)	772 (20.2%)			
Middle	901 (19.3%)	719 (20.5%)			741 (19.4%)	719 (18.8%)			
Upper middle	999 (23.9%)	809 (24.1%)			795 (20.8%)	809 (21.2%)			
Upper	1008 (24.4%)	784 (23.7%)			764 (20.0%)	784 (20.5%)			
Education									
Elementary school or less	1251 (19.6%)	992 (18.2%)	0.01	0.2	997 (26.1%)	992 (26.0%)	0.02	0.4	−35.1
Middle school graduate	610 (10.8%)	489 (11.2%)			493 (12.9%)	489 (12.8%)			
High school graduate	1520 (34.6%)	1156 (33.3%)			1217 (31.9%)	1156 (30.3%)			
College or higher	1412 (35.0%)	1181 (37.3%)			1111 (29.1%)	1181 (30.9%)			
Occupation									
Managers, experts, and related worker	469 (11.7%)	443 (14.0%)	−0.06	0.03	385 (10.1%)	443 (11.6%)	−0.02	0.2	72.1
Office worker	401 (10.3%)	302 (10.1%)			337 (8.8%)	302 (7.9%)			
Service and sales worker	556 (12.8%)	444 (12.4%)			469 (12.3%)	444 (11.6%)			
Skilled agricultural, forestry, and fishery workers	227 (3.7%)	213 (4.4%)			182 (4.8%)	213 (5.6%)			
Technician, machine operator, and assembly worker	457 (11.9%)	416 (12.9%)			407 (10.7%)	416 (10.9%)			
Simple labor worker	595 (10.9%)	409 (9.5%)			462 (12.1%)	409 (10.7%)			
Unemployed (housewife, student, etc.)	2088 (38.8%)	1591 (36.7%)			1576 (41.3%)	1591 (41.7%)			
Alcohol Intake									
Non-drinker in the past year	1079 (21.5%)	847 (20.9%)	−0.01	<0.001	852 (22.3%)	847 (22.2%)	-0.01	0.2	-46.6
Less than once a month	888 (18.8%)	662 (17.4%)			653 (17.1%)	662 (17.3%)			
About once a month	423 (9.4%)	329 (9.4%)			327 (8.6%)	329 (8.6%)			
2–4 times a month	815 (18.3%)	640 (17.9%)			699 (18.3%)	640 (16.8%)			
About 2–3 times a week	548 (13.0%)	556 (16.6%)			485 (12.7%)	556 (14.6%)			
4 or more times a week	276 (6.0%)	282 (8.0%)			251 (6.6%)	282 (7.4%)			
Never drank alcohol	764 (13.0%)	502 (9.8%)			551 (14.4%)	502 (13.1%)			
Smoking History									
Nonsmoker	3073 (60.0%)	2157 (51.6%)	0.13	<0.001	2229 (58.4%)	2157 (56.5%)	0.02	0.08	86.8
Current smoker	654 (16.8%)	687 (20.6%)			598 (15.7%)	687 (18.0%)			
Ex-smoker	1066 (23.2%)	974 (27.9%)			991 (26.0%)	974 (25.5%)			
Activity Limitation due to Dementia									
Yes	3 (<1%)	6 (<1%)	<0.01	0.9	3 (<1%)	6 (<1%)	<0.01	0.4	16.9
No	4790 (99.9%)	3812 (99.9%)			3815 (99.9%)	3812 (99.8%)			

^1^ Categorical variables were presented as actual numbers (N) with corresponding weighted percentages (%), whereas continuous variables were reported as weighted means ± weighted standard deviations (SD). ^2^ SMD: standardized mean difference, PBR: percentage of bias reduction. ^3^ For statistical analysis, Rao–Scott χ^2^ test was employed for categorical variables, and the complex sample *t*-test was used for continuous variables.
